# Computed tomography and magnetic resonance imaging of a plexiform angiomyxoid myofibroblastic tumor: a case report

**DOI:** 10.1186/s12880-017-0180-1

**Published:** 2017-01-19

**Authors:** Hiroyuki Akai, Shigeru Kiryu, Masaru Shinozaki, Yasunori Ohta, Yoshiyasu Nakano, Koichiro Yasaka, Kuni Ohtomo

**Affiliations:** 1 Department of Radiology, Institute of Medical Science, University of Tokyo, 4-6-1 Shirokanedai, Minato-ku, Tokyo, 108-8639 Japan; 2 Department of Surgery, Institute of Medical Science, University of Tokyo, 4-6-1 Shirokanedai, Minato-ku, Tokyo, 108-8639 Japan; 3 Department of Pathology, Institute of Medical Science, University of Tokyo, 4-6-1 Shirokanedai, Minato-ku, Tokyo, 108-8639 Japan; 4 Department of Radiology, Graduate School of Medicine, University of Tokyo, 7-3-1 Hongo, Bunkyo-ku, Tokyo, 113-8655 Japan

**Keywords:** Stomach, Mesenchymal tumor, Magnetic resonance imaging, Computed tomography

## Abstract

**Background:**

Plexiform angiomyxoid myofibroblastic tumor (PAMT) is a very rare mesenchymal tumor of the stomach. Here we report a case of pathologically confirmed PAMT with an unique cyst formation.

**Case presentation:**

A 55-year-old male with a 10-year history of a gastric subepithelial tumor underwent computed tomography (CT) and magnetic resonance imaging (MRI). Two cysts were observed in the tumor, and the cyst wall showed moderately high intensity on T2-weighted images compared with the gastric wall. On dynamic study, the cyst wall showed a gradual enhancement pattern, and prominent enhancement was observed in the delayed phase. Laparoscopic partial gastric resection was performed, and a pathological diagnosis of PAMT was rendered.

**Conclusion:**

We present a rare case of gastric PAMT, which was uniquely presented as cysts. One of the cysts in the tumor had an epithelial wall lining, which had never been reported before in gastric mesenchymal tumor, in addition to partial glandular structure. We reviewed our case, focusing on radiologic-pathologic correlation, and suggested hypothesis of cyst formation. According to our findings, PAMT with cyst formation would be included of differential diagnosis of gastric subepithelial tumors.

## Background

Plexiform angiomyxoid myofibroblastic tumor (PAMT) is a rare mesenchymal tumor of the stomach that was first described by Takahashi et al. in 2007 [1]. The incidence of PAMT is estimated to be less than 1 in 150 compared with gastric gastrointestinal stromal tumor (GIST) [2], and only 34 cases of PAMT have been reported in the English language literature, with scant data pertaining to computed tomography (CT) and magnetic resonance imaging (MRI) findings [3–19]. Here, we describe a case of PAMT with unique cystic formation, focusing on the imaging findings and radiological-pathological correlation.

## Case presentation

A 55-year-old male with a 10-year history of a gastric subepithelial tumor followed-up by upper gastrointestinal endoscopy at other clinic visited our institution with a complaint of tumor enlargement. The patient did not experience any discomfort, and his laboratory test results were all normal.

Upper gastrointestinal endoscopy showed a subepithelial mass, 2 cm in diameter, at the posterior wall of the gastric angle. Small dimples were observed at the apex of the tumor. A biopsy was performed, and viscous liquid was ejected from the tumor. The biopsy results were insufficient for a diagnosis.

**Fig. 1:**
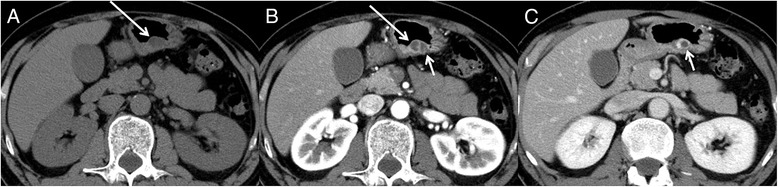
Dynamic enhanced computed tomography (CT) (**a**, non-enhanced CT; **b**, arterial phase; **c**, delayed phase) revealed a gastric subepithelial tumor at the posterior wall of the gastric angle. Long arrow indicates the cyst shrank, and short arrow indicate the cyst enlarged on the follow-up CT

On non-enhanced CT (Aquilion One; Toshiba, Tochigi, Japan), 19 mm subepithelial tumor showed low attenuation. Dynamic enhanced CT was performed after injection of 100 mL iomeprol 350 (Bracco-Eisai, Tokyo, Japan) at a rate of 3.3 mL/s. The arterial phase was obtained at 30 s, and the delayed phase was obtained at 90 s. The tumor showed two cysts and a thin cyst wall with moderate enhancement in the arterial phase and strong enhancement in the delayed phase (Fig. 1). On follow-up CT performed 4 months later for preoperative evaluation, the whole tumor slightly enlarged to 23 mm, and one of the cysts was enlarged (Fig. 1 short arrow), whereas the other showed shrinkage (Fig. 1 long arrow). No lymphadenopathy was seen.

**Fig. 2:**
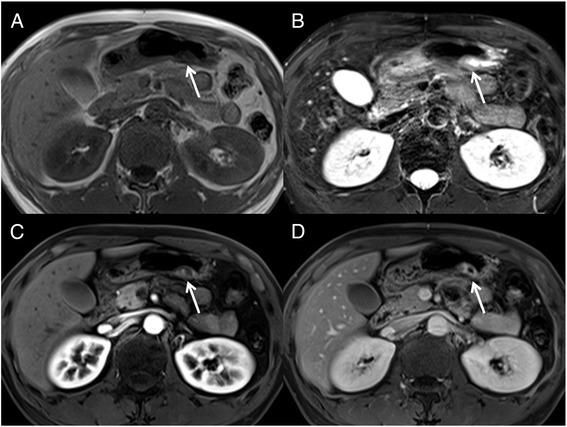
Magnetic resonance imaging (MRI) (**a**, T1-weighted image; **b**, fat suppressed T2-weighted image; **c**, arterial phase; **d**, delayed phase) of the tumor showed cysts with a thin wall. The wall exhibited hyperintensity on T2-weighted images and a gradual enhancement pattern, which reflects the myxoid nature of the tumor

On MRI (MAGNETOM Skyra 3 T; Siemens, Erlangen, Germany) performed 4.5 months later from the initial CT scan, 2 cm subepithelial tumor composed by two cysts was also observed. The cyst showed high intensity, and the cyst wall showed moderately high intensity, compared with the gastric wall, on T2-weighted images. On T1-weighted images, the cyst showed low intensity, and the cyst wall showed iso-intensity to the gastric wall. On diffusion-weighted imaging (image not shown), no apparent diffusion restriction was seen, and the tumor showed iso-intensity to the gastric wall. Dynamic enhanced MRI was performed after injection of 0.2 mL/kg body weight gadopentetate dimeglumine (Magnevist; Japan Schering, Osaka, Japan) at a rate of 2 mL/s. The arterial, portal and delayed phases were obtained at 20, 50 and 120 s, respectively. As with the dynamic enhanced CT findings, the cyst wall showed a gradual enhancement pattern, with strong enhancement seen in the delayed phase (Fig. 2).

The differential diagnosis of the gastric subepithelial tumor included schwannoma, GIST, ectopic pancreas, duplication cyst with cyst wall inflammation, and adenocarcinoma arising from a duplication cyst. Although the patient had a long history of a tumor, laparoscopic partial gastric resection was performed, because the possibility of malignancy could not be ruled out.

**Fig. 3:**
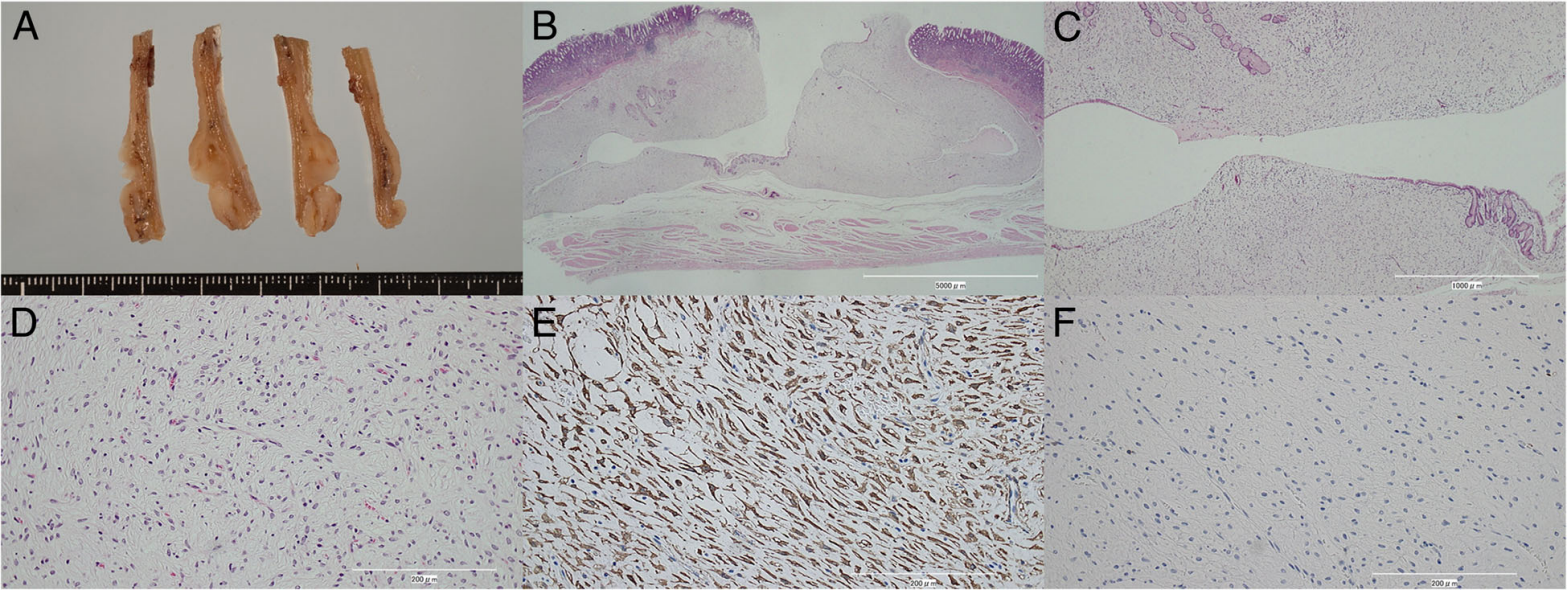
Pathological findings of the tumor. **a**, macroscopic findings: the tumor is located mainly in the submucosa, and two cysts are observed within the tumor; **b**–**d**, microscopic examination (hematoxylin and eosin staining on three different power fields) showing a partial epithelial lining in the cyst wall and some glandular structures within the tumor. Spindle tumor cells are seen in the rich myxoid stroma. The tumor cells are positive for alpha-smooth muscle actin (**e**) and negative for c-KIT (**f**)

Macroscopically, a 17-mm yellowish nodular lesion was present from the lamina propria mucosa to the submucosa. Two cysts were observed within the tumor, one of which showed an epithelial wall lining. The tumor also showed a partial glandular structure. Histologically, the tumor cells were bland and spindle shaped, without significant nuclear atypia or mitosis, accompanied by abundant myxoid matrix. In the myxoid matrix, numerous small, thin-walled vessels were observed. Immunohistochemically, the tumor cells were positive for alpha-smooth muscle actin and negative for c-KIT, CD10, CD34, desmin, S-100 protein, and epithelial membrane antigen. Based on the above results, the tumor was diagnosed as PAMT (Fig. 3). MIB-1 labeling index was less than 1%. Due to this pathological result, no further follow up is performed.

## Discussion

PAMT of the stomach is a rare mesenchymal tumor that typically shows a multinodular plexiform growth pattern. PAMT is composed of myofibroblastic spindle cells, separated by intercellular myxoid or fibromyxoid matrix, with rich arborizing capillaries [1]. Immunohistochemically, PAMT is positive for vimentin and muscle actin [10]. c-KIT, CD34, and S-100 protein is negative in PAMT, which are usually positive in GIST, GIST and solitary fibrous tumor, and schwannoma and neurofibroma, respectively. Typically, PAMT is positive for alpha-smooth muscle actin, and focal immunoreactivity for desmin and caldesmon is occasionally seen [10].

PAMT may also contain tumor cells with fibroblastic or smooth muscle characteristics [2, 4, 16] and therefore is sometimes reported as “plexiform fibromyxoma” or “plexiform angiomyxoid tumor” [2, 4]. However, Sing et al. suggested that PAMT could be distinguished from plexiform fibromyxoma according to its immunohistochemical and clinicopathological features [9]. Those authors claimed that vascular invasion and extragastric extension are usually seen in plexiform fibromyxoma, with no immunoreactivity against caldesmon or desmin; in contrast, PAMT does not show vascular invasion, and focal reactivity for desmin and caldesmon is seen occasionally. Takahashi et al., who discovered PAMT, suggested that plexiform fibromyxoma is a fibroblastic trait of PAMT [10]. Although the precise nomenclature remains controversial, “plexiform fibromyxoma” was classified as a mesenchymal tumor of the stomach in the 2010 World Health Organization classification of digestive system tumors [20]. Because the majority of tumor cells show a predominantly myofibroblastic nature, we believe that PAMT is the appropriate diagnostic term reflecting the histogenesis and histology of this tumor. Furthermore, we believe that this is supported by the fact that more cases have been reported as PAMT, rather than as plexiform fibromyxoma, even after 2010.

To date, including the present case, 35 cases of PAMT have been reported in the English language literature. Of these cases, the patient age ranged from 7–75 years (median = 43 years), and no sex difference was evident (M:F = 17:18). Tumor size varied from 1.5 to 15 cm (mean 5.2 cm), and the majority of the PAMTs occurred in the gastric antrum and pylorus. However, PAMTs can occur anywhere in the stomach, including the fundus and body [7, 12, 15]. Since mucosal ulceration was seen in approximately half of the cases [1, 2, 4–6, 11, 12, 15, 17], PAMT may cause hematemesis, gastrointestinal bleeding, and anemia, although incidentally found PAMTs are not rare [9, 12, 15, 16]. Although vascular invasion was seen in some of the cases reported by Miettinen et al. [2], the prognosis of PAMT seems to be benign tumor, since no recurrence or metastasis has been reported to date.

There are only a few studies reporting CT and MRI findings of PAMT (summarized in Table 1). Among the cases in which dynamic studies were performed, one showed a gradual enhancement pattern, and three showed a gradual enhancement pattern plus prominent enhancement in the delayed phase, which is compatible with the myxoid nature of the tumor. In the case who demonstrated a gradual enhancement pattern, there was only relatively weak enhancement in the delayed phase [18]. This might be because the tumor was negative for alpha-smooth muscle actin, showing mainly the fibroblastic traits of PAMT. A case reported by Kang et al. also showed strong enhancement, although the exact timing of performing CT scanning was not specified [12]. Only one case, reported by Sing et al., showed poor enhancement [9]. The CT image shown in their paper was apparently taking during the arterial phase, which may have been too early to demonstrate enhancement of this myxoid tumor. Including our case, there are only two reports of PAMT that include MRI findings [18]. Both cases showed hyperintensity on T2-weighted images and no apparent diffusion restriction. Along with the findings of the dynamic study, these MRI findings reflect the myxoid nature of this tumor. Furthermore, we assume that prominent enhancement in the delayed phase is also due to rich small vessels in the myxoid matrix. Although these are characteristic imaging findings, it may be difficult to distinguish PAMT from other myxoid tumors, such as myxoid variants of GIST.

**Table 1:** Radiological feature of 6 cases of PAMT

Case	Age/Sex	Tumor size (cm)	Tumor location	Unenhanced CT	T1WI (MRI)	T2WI (MRI)	DWI (MRI)	Dynamic CT or MRI	Reference
1	35/F	4	antrum	NA	NA	NA	NA	EC(poorly enhancing)	[9]
2	47/M	3	mid body	NA	NA	NA	NA	EC(prominent enhancing)	[12]
3	38/F	3.5	upper body	NA	NA	NA	NA	PE	[15]
4	27/F	4.6	antrum	inhomogeneous low	NA	NA	NA	PE	[17]
5	60/M	2.0	antrum	NA	iso	high	not high	gradual enhancement	[18]
Present case	55/M	1.7	angle	low	iso	high	iso	PE	

*NA* indicates that data was not available, *EC* indicates that only enhanced, *CT* was performed and parenthesis shows description in original case reports, *PE* represents gradual enhancement pattern plus prominent enhancement in delayed phase

For T1WI, T2WI and DWI, tumor signal is compared to that of the gastric wall

Cyst formation appears to be rare in PAMT, and only three cases have been reported previously. In two of those cases, the tumor was continuous and showed a sac-like structure that had a hemorrhagic inner wall with multiple perforations [8, 14]. In the other case, hemorrhage, necrosis, and cystic degeneration were seen in a tumor 14 cm in size [10]. Unlike these former cases, unique cyst formations were observed within the tumor in our case. Of the two cysts, the shrunken cyst did not show an epithelial wall lining, while the enlarged cyst did. We speculate that both cysts had an epithelial lining, but that the lining in the shrunken cyst was exfoliated. The cause of these cysts is unclear, and two explanations can be offered: the first is that the PAMT grew until it surrounded the heterotopic epithelial cells, and a cyst formed from the mucus secreted from these cells. This hypothesis may be supported by the fact that the tumor showed a partial glandular structure. The other explanation is that the PAMT occurred near these cysts and then grew until it surrounded them. Our case is the first report of a gastric subepithelial mesenchymal tumor containing a cyst with an epithelial lining; therefore, more data are required to confirm the true nature of these cysts.

## Conclusion

In conclusion, we reported a case of PAMT, a rare subepithelial mesenchymal tumor of the stomach. MRI and dynamic study findings reflected the myxoid nature of this tumor well. A cyst with an epithelial wall lining was observed in the gastric subepithelial mesenchymal tumor for the first time. We believe radiologist should be aware that PAMT should be included to differential diagnosis of cystic subepithelial tumor with myxoid imaging feature.

## Abbreviations

CT: Computed tomography
GIST: Gastric gastrointestinal stromal tumor
MRI: Magnetic resonance imaging
PAMT: Plexiform angiomyxoid myofibroblastic tumor
